# Application of Sensors for Incorrect Behavior Identification in a Transport System

**DOI:** 10.3390/s23031635

**Published:** 2023-02-02

**Authors:** Martin Mantič, Jozef Kuľka, Robert Grega, Matúš Virostko, Melichar Kopas

**Affiliations:** Faculty of Mechanical Engineering, Technical University of Kosice, 040 01 Košice, Slovakia

**Keywords:** overhead crane, crane skewing, signal measurement

## Abstract

This article focuses on cranes that are moving on a fixed crane track. There are specific problems and malfunctions arising during the operation of these cranes caused mainly by the crane skewing phenomenon. Crane skewing induces undesirable additional forces as a result of force contact between the crane wheel flange and the head of the crane track rail. This negative phenomenon induces additional stress in the crane construction as well as wear of the crane components and, finally, a global reduction of the crane operational durability and reliability. There is described in this article a methodology and data processing for the experimental measurement targeted on the crane skewing, namely in the case of a bridge crane installed in a laboratory. The crane skewing phenomenon was experimentally induced by an intentional disruption of speed synchronization of the crane travel drives. The intensity of the crane skewing was measured by means of the strain gauge sensors. A suitable application of the measuring sensors, together with the utilization of the drive control algorithm, enables efficiency to eliminate crane skewing and also prevent its occurrence.

## 1. Introduction

Transport, conveying and material handling are the basic activities performed in almost all manufacturing industries, i.e., from the processing of the raw material to the distribution of the final products. These activities are provided using suitable transport and handling technology, in which the lifting equipment plays a central role. One wide range of such equipment consists of overhead (bridge cranes). These cranes generally belong to the transport and handling machinery, which allows the movement of material, usually within a rectangular handling area. The bridge cranes are the basic lifting and handling machines used in production halls, plants, warehouses, transshipment yards, etc. They are characterized by the fact that their steel supporting structure consists of a crane bridge, which usually moves along a raised crane runway, i.e., along a two-rail crane track. A crane trolley, or possibly a crane hoist with a crane hook, moves on or under the crane bridge. The bridge cranes are the typical representatives of cyclically operating transport and handling machines. However, their operation is subjected to various undesirable influences, which negatively affect their service life and technical reliability.

A significant negative aspect of the operation of bridge-type cranes is the technical problems associated with the wear of the crane wheels and crane track, whereby this wear causes crane skewing [1]. Crane skewing induces horizontal forces between the crane and the crane track during the motion of the overhead traveling crane on the crane track. The main causes of crane skewing include unevenness of the crane track, unequal loading of the traction drives depending on the position of the crane trolley, slips and different sizes of travel wheels, acceleration or braking of the crane and combinations of these causes [1,2]. Elimination of these negative impacts can be realized in different ways. For example, authors in [3] present a construction design and results of laboratory tests of the so-called mechanical stress detectors, which can be used to detect deformation of the steel structure of a bridge crane caused due to the effect of crane skewing. The paper [4] describes how the dual-motor driven bridge cranes should be automatically operated using a skew controller, which was successfully simulated in MATLAB. Krupiarz P. [5] presents a case study of a fuzzy logic controller equipped with four inputs and one output. This regulator implements an anti-skewing control determined for a semi-gantry crane. The control system is suggested in order to monitor two analog approach sensors, measure the distance between the crane trolley and the crane track rail, as well as to offer torque feedback from the drives. Wang in [6] presents a novel dynamic sliding mode variable structure control algorithm. The proposed controller is able to ensure global stability of the crane drives in the time interval for different crane loading and for various frictional resistances. In a paper elaborated by Streltsov et al. [7], the brake system of the crane is monitored as the cause of uneven braking and, thus, also as the cause of different rotations of the crane driving wheels, which generate skewing of the crane. The kinematic scheme is, in principle, based on the distribution of energy flows. Ho et al. [8] propose nonlinear optimal control schemes in order to reduce transmission time, energy consumption and vibration for the process of crane system load. The new idea, in this case, consists of the application of an electromechanical clutch determined for intelligent disengagement of connection between the motor and the crane load during movement.

Causes of skewing and the calculation of the forces that arise during skewing are presented, for example, in the contribution [9]. Other examples are introduced in the publications [10,11], where the authors discuss an experimental determination of forces acting on the bridge crane wheels. The analysis of skewing and its causes is described in [12], and the resulting failures are analyzed, e.g., in [13]. Many authors perform analyses of the crane, such as dynamic models, which generally describe the behavior of the crane during acceleration [14] and enable the determination of the horizontal transversal forces. The basic assumption of these models is that there is no contact between the wheel flange and the rail of the crane track. In the case of such models, it is not possible to define the initial pre-stress before starting the crane. In fact, the pre-stress is generated as a result of the previous crane travel, and it also remains acting after the crane is stopped. There are determined in the contribution from [15] forces arising between the crane and the crane track by means of motional equations that correspond to the degree of freedom in the dynamic model. The authors in [16] analyzed the correct choice of the crane cross-section geometry, taking into consideration the stiffness of the crane structure itself, as well as regards the mutual connection of the individual constructional parts. Hoang [17] presents a dynamic model with six degrees of freedom. Based on the criteria of Lyapunov and Barbalat, the convergence of tracking errors is ensured. The given model also monitors vertical vibrations, which have serious negative impacts on the overall durability of the crane structure. The suitability of this model and the effectiveness of the applied control algorithms are verified using numerical simulations performed on a real crane. Fidrovska et al. [18] are investigating the dynamic effect of the crane wheel impact on the jointing point of the rails and also the overall response of the bridge crane steel structure to this case of load at different crane trolley positions. This loading is compared with the values of the dynamic coefficient, which is used in dimensioning the crane-supporting structure.

The team of authors disposed of a laboratory overhead traveling crane, which was installed at their workplace. Based on the analysis of the used procedures and considering the suitability of the model for the given experiment, it applied a proper method intended for the elimination of crane skewing. The principle of this method consists of the control of the crane drives, while the occurrence and extent of the crane skewing were evaluated by experimental measuring using the strain gauge sensors. The whole experimental procedure is presented and described in the following chapters.

## 2. Theoretical Description

There are many technical ways or methods whose task is to ensure smooth and direct travel of the bridge cranes. However, in most cases, especially from an economic point of view, methods have mainly been used that deal with the consequences of undesirable phenomena but not their origin. The skewing of bridge cranes that are moving along a fixed crane track is one of the main negative phenomena in the operation of these lifting devices. Assuming an ideal crane track, this is a situation where one side of the crane does not move identically relative to the other side, which causes the wheels to friction against the crane track and consequently their gradual wear. The cause of this friction is the emergence of undesirable horizontal forces.

**Figure 1:**
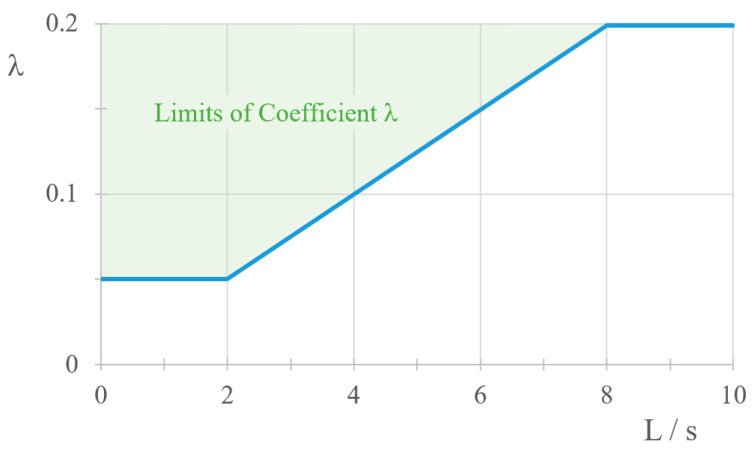
Values of the λ coefficient.

The technical standard, which is valid for the design of the crane steel structures [19], states that crane skewing occurs when two crane travel wheels are traveling on the crane track rail. The skewing of the crane is computationally characterized in the form of a pair of forces acting perpendicular to the rail at the level of its upper edge. The forces of this pair are determined according to the next equation:

H_tp_ = λ ΣK (1)

where λ = 0.025 L/s (but not less than 0.05 and not more than 0.2). The value of λ can also be read from the diagram in Figure 1,

L—is the span of the crane,

s—is the wheelbase of the crane wheels,

ΣK—is the load acting on the wheels on the more loaded side of the crane track from the self-weight of the crane and from the crane trolley weight with the load situated in the most effective position [19].

**Figure 2:**
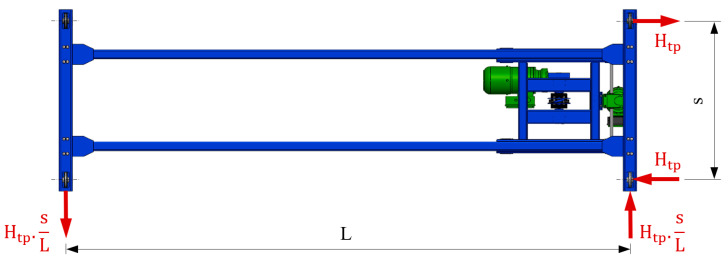
Action scheme of forces caused by crane bridge skewing. H_tp_—force causing the crane skewing, L—span of the crane, s—wheelbase of the crane wheels.

Figure 2 presents an action scheme of forces caused by crane bridge skewing.

The most common causes of uneven operation of the crane include:

- crane track not constructed in accordance with the key specifications and tolerances;
- incorrect geometry of the crane, inaccuracies in the manufacture of its structure and the laying of the crane track, and so on;
- slippage of the crane drive wheels due to an incorrectly designed drive train, unevenness or contamination of the crane track;
- uneven operation of the driving motors if it is a separate type of drive. At the same time, due to even minimal deviations in the speed of the wheels, the attendance to a gradual deflection from direct travel and other consequences is its skewing operation. If it is a drive with a continuous shaft, in this case, uneven twisting of the drive shaft may occur;
- uneven load on the drive wheels. It arises mainly by the position of the crane trolley on the crane, where, due to the lightening of one drive wheel and the load on the other, there is a difference in the speed of the wheels and thus a deflection from the direct travel of the crane;
- others.

**Figure 3:**
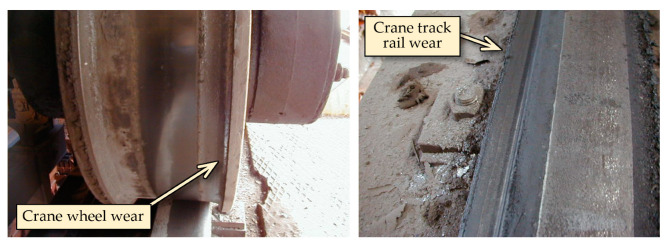
Examples of wear on the crane wheels and crane track rails.

Unfavorable operational conditions arising during the operation of the crane with skewing are causing mainly wear of the crane wheels, where the skewing leads to a gradual loss of material from wheel flanges as well as a loss of material on the head of the crane track. In Figure 3, there are visible examples of crane wheel wear and crane track rail wear that come out in practice.

The crane skewing not only decreases the crane’s service life but the overall impact is also on the crane’s structural parts, such as the support of the crane wheels. Here, additional stresses can be noted on the bearings, causing the need for premature replacement. An accompanying phenomenon when skewing is also an unpleasant acoustic sound caused by dry friction of the crane wheels against the crane track, which, however, can have adverse effects on the health or well-being of workers in a given operation.

## 3. Simulation Analysis

**Figure 4:**
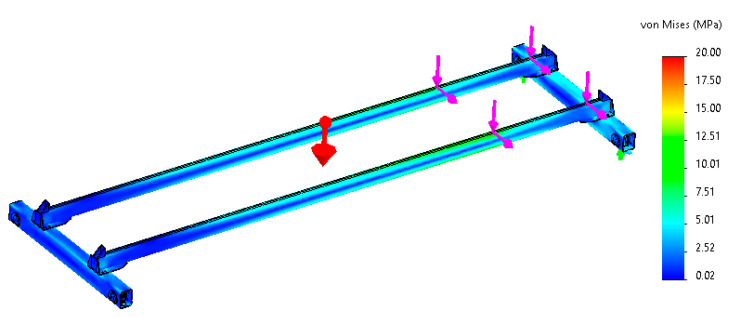
Crane bridge simulation model.

Experimental measurements were preceded by a thorough simulation analysis of the crane bridge in order to get an idea of how this structure behaves during crane skewing. The simulations were run on a computational 3D model using the finite element method (FEM). This model was created according to the shape and dimensions of the real crane installed in the laboratory. The aim was to identify places on the bridge structure that show increased values of the tensile and pressure stresses. These locations are then suitable for the application of the sensors determined for the identification of the crane skewing in experimental measurements. A view of the simulation model is shown in Figure 4.

**Figure 5:**
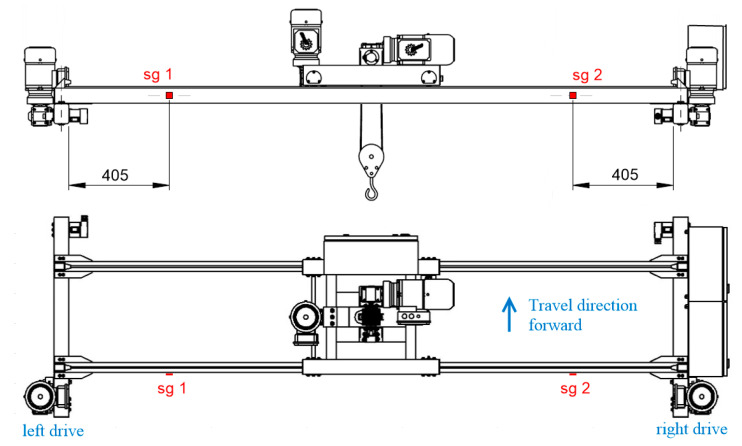
Placement of the strain gauge sensors on the crane bridge construction.

The values of the acting loads caused by the crane trolley were entered from the experimentally measured values of wheel pressures. In the simulation model, the horizontal inertial effects of forces in the direction of movement of the crane bridge were also taken into account. However, when determining the actual measuring points, it was also necessary to consider the technological and production aspects of the whole crane construction. The aim was to avoid the locations of joints, welds, as well as other components that could affect the accuracy of the experimentally measured data. After considering all aspects, there were proposed measuring points sg1 and sg2 that are illustrated in Figure 5.

**Figure 6:**
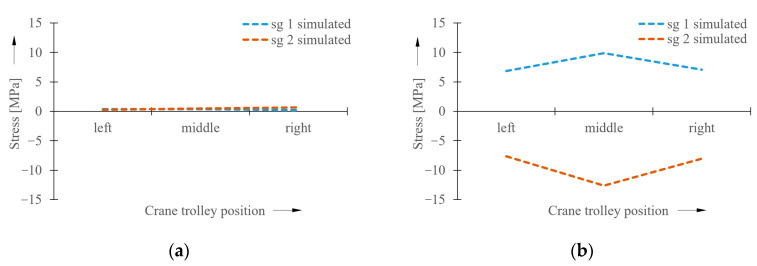
Stress values obtained from simulation models. (**a**) without crane skewing, (**b**) with forced crane skewing.

Simulations of the crane’s operational states were performed under various operational conditions and using various load cases. This article presents analyses of the crane bridge when traveling without crane skewing and with forced crane skewing. In both cases, the used load with a weight of 50 kg was in three different positions of its action. The first case simulated a condition corresponding to the position of the crane trolley with the load on the left side of the crane bridge. The second case was the loaded crane trolley position in the middle of the crane bridge span, and the third was on the right side of the crane bridge. The stress values obtained from the simulation models at the measuring points sg1 and sg2 are shown in the graph in Figure 6.

## 4. Experimental Identification of Bridge Crane Skewing

**Figure 7:**
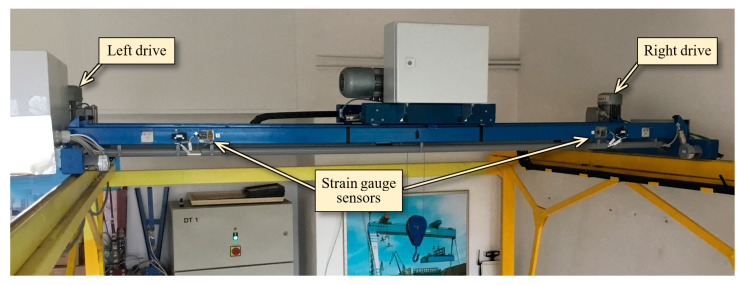
View of the bridge crane installed in the laboratory.

Identification of the crane skewing causes was carried out on the in-laboratory installed double-girder bridge crane (Figure 7), the span of which is 2.5 m, and the load capacity is 50 kg.

Various measurement methods were applied to identify the causes of the skewing formation, but the most suitable was the application of the strain gauge sensors. The strain gauge sensors identify skewing, and they also provide information about the intensity of skewing. Two strain gauge sensors were applied, which in Figure 5 are marked as “sg 1” and “sg 2,” also with the determination of their exact position. The strain gauge sensors were applied to the neutral axes of the cross-sections of the main girders and cross beams in order to eliminate the effect of bending. This positioning ensured the measurement of normal stress increments that are caused only by the crane skewing in the horizontal plane.

**Figure 8:**
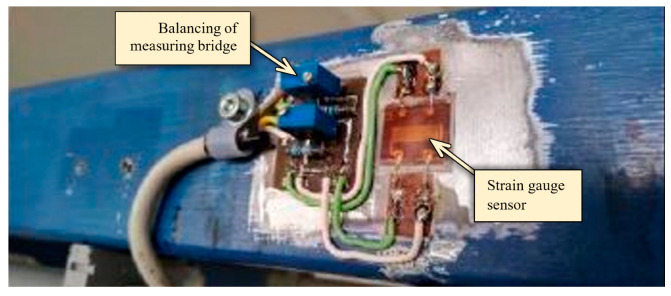
Application of the strain gauge sensors on the given bridge crane.

For the purpose of the experimental measurements, the sensors of type HBM 1-XY91-10/350 were used. The electrical wiring of strain gauge sensors was realized as a whole strain gauge Wheatstone bridge with automatic temperature compensation. With regard to the utilization of the whole measuring range, a special device was designed for hardware zeroing of the strain gauge sensors using the electronic trimmers, see Figure 8.

Transformation of the resistance changes from the strain gauge sensors to the output signal was performed by the EMS170 converters from the EMSYST company.

**Figure 9:**
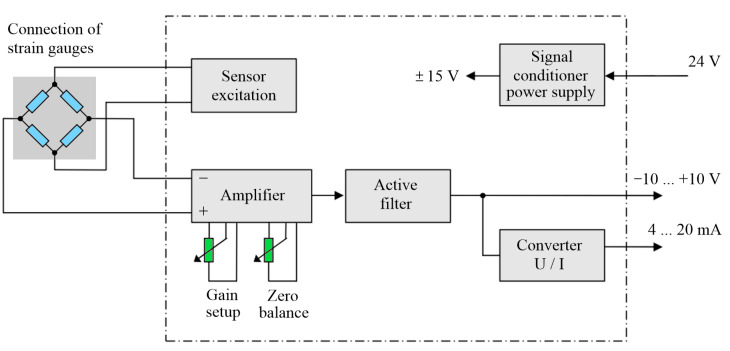
Block diagram of the EMS170 converter.

These converters supply the sensors with stabilized voltage and also with direct current, and they amplify the output signal. The block diagram of the EMS170 converter is displayed in Figure 9.

The experimental measurement itself was carried out during the forced skewing of the crane. The forced crane skewing was realized by changing the output frequency of one of the frequency converters to a higher value. Due this asymmetrical change of output frequency one side of the crane was moving faster than second side along the crane track. Thus, the crane deviated from its direct travel, which induced the crane skewing. The difference in output frequencies was 7 Hz, which corresponded to a difference in circumferential speeds of 1.557 m/min. The experimental measurements were made at different positions of the crane trolley on the crane bridge and with different load weights.

**Figure 10:**
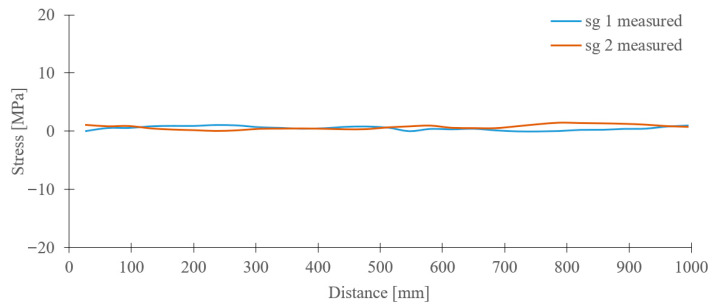
Course of stress values in the crane bridge construction during traveling of the crane without skewing.

The results of experimental measurements, which are presented in Figure 10, were obtained during the travel of the crane along the crane track with the crane trolley position in the middle of the crane bridge and with the load of 50 kg, i.e., the ideal operational condition, without skewing of the crane.

**Figure 11:**
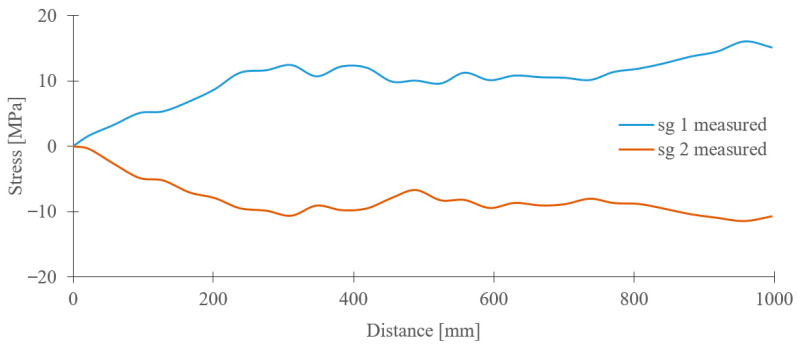
Course of stress values in the crane bridge construction during traveling of crane with forced skewing.

Figure 11 illustrates the course of the stress values obtained from the strain gauge sensors during a similar journey of the crane along the crane track but with a forced skewing on the left side, i.e., using the increase in the frequency of the driving motor near the sg1 sensor.

It is visible from Figure 11 that the course of the tensile and pressure stresses in the crane bridge construction is caused due to increasing the transverse forces during the non-synchronous movement of the driving motors. The experimental measurement demonstrates a sufficient sensitivity of the strain gauge sensors to identify the crane skewing as well as to determine its intensity. At the same time, these data also provide information about the additional loading of the whole crane bridge construction.

**Figure 12:**
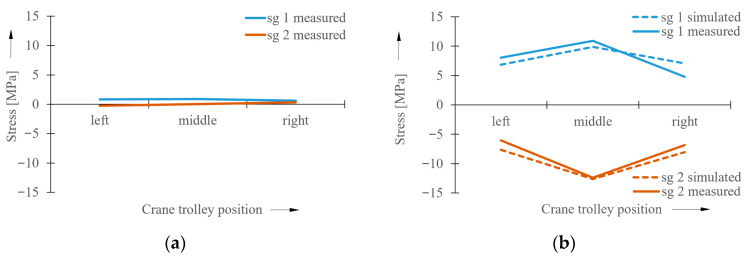
Experimentally measured stress values in crane bridge structure. (**a**) without crane skewing, (**b**) with forced crane skewing.

The maximum values of the measured stresses in the bridge structure are summarized in the graphs in Figure 12.

## 5. Discussion

**Figure 13:**
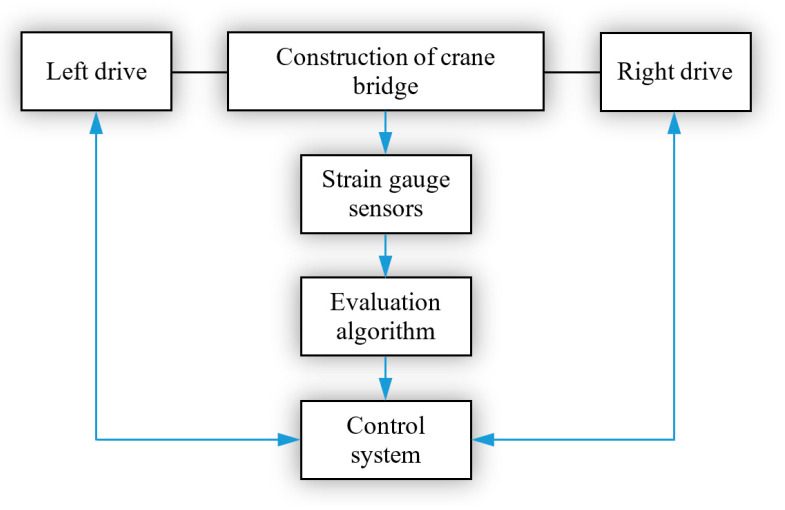
Measuring chain proposed for elimination of crane skewing.

A skewing elimination system was designed for the installed strain gauge sensors, which is based on the change in the values of the normal stress increments obtained from these sensors. When the changes of these values are above the limit value, which represents the limit of the skewing beginning, the speed of crane wheel rotation is modified by means of the proposed control system. These changes are achieved by a smooth change in the speed of the driving motors on the left and right sides of the crane bridge using the frequency converters. This intervention will gradually decrease the values of measured increments of the normal stresses as well as decrease the values of horizontal transverse forces. As a result, the angle of crane skewing is reduced, the crane gradually returns to its direct travel, and thus the deflection of the crane bridge is eliminated. The measuring chain proposed for this method of skewing elimination is illustrated in Figure 13.

**Figure 14:**
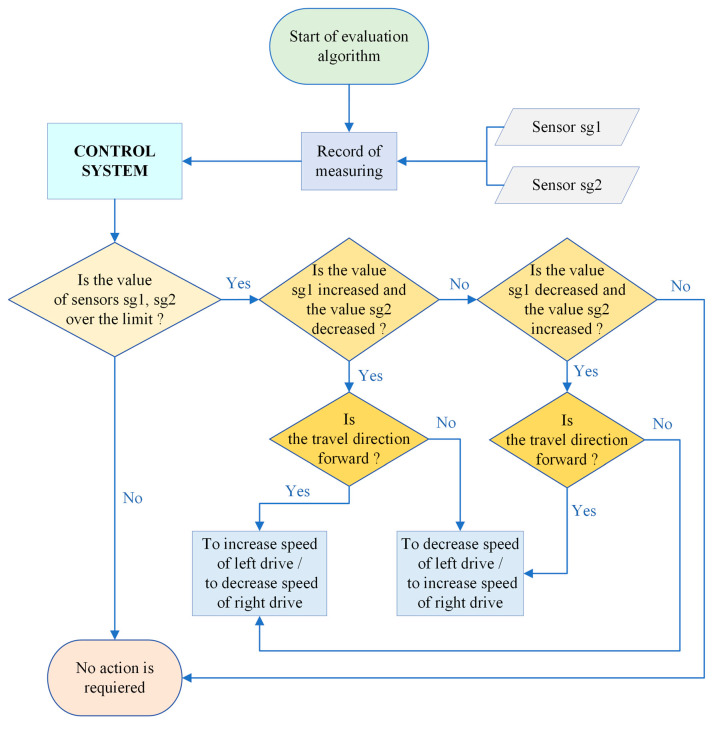
Evaluation algorithm determined for elimination of the crane skewing.

The proposal of the evaluation algorithm determined for the elimination of the crane skewing, created in the form of a flowchart, is presented in Figure 14.

It follows from the experimentally measured values of the normal tensile stresses and from the measurement of directional deviations of the wheel flanges relative to the crane track rail on the laboratory bridge crane that, in this case, the limit stress value relating to the occurrence of the crane skewing is 1.5 MPa. This limit value is important for the evaluation algorithm intended for this bridge crane. The measurement of directional deviations served to determine the time moment when the state of contact between the wheel flange and crane track rail occurs. At this time, pure crane skewing occurs, and the influence of other horizontal forces acting on the crane structure is eliminated during the measurements. It is necessary to note that the stress value, which is decisive for the occurrence of crane skewing, has to be determined individually for each crane. The graph in Figure 12b compares the results obtained from simulation analyses with the values from experimental measurements in the case of the analyzed laboratory crane. The obtained results are in very good mutual conformity, which confirms the correctness of the simulation models. Therefore, it is possible to say that this simulation model is suitable for utilization in all bridge-type cranes. A well-created simulation model can ensure the faster determination of the input values that are required for the control algorithm determined for the elimination of the crane skewing.

## 6. Conclusions

With the development of new technologies, lifting equipment has also evolved over time, and electronic systems, sensing technology and modern communication technologies have also been added to their control. This significantly contributes to the possibility of eliminating various undesirable conditions (such as crane skewing, swinging of the load, etc.) that may arise during their operation and, consequently, to increasing their service life and reliability. The application of, for example, appropriately positioned strain gauge sensors on the crane bridge structure can be easily used to identify the occurrence of skewing and its intensity in the case of bridge cranes. The output data obtained from the sensors, together with other information about the current position of the crane on the crane track, offer a powerful tool for the subsequent elimination of the crane skewing process by timely intervention in the control of the crane travel drives.

There are several ways to identify crane skewing based on the strain gauge sensors (e.g., [7]), where additional sensing elements are applied to the crane structure. In our case, the strain gauge sensors are directly glued to the structure in the exactly defined points in the neutral axis of the cross-section of the beam. Such application methods of the sensors can be more sensitive to external loads during the operation of the crane.

The stress value, which represents the limit of the crane skewing occurrence, is an important input into the control system determined for the elimination of the crane skewing in our case. This stress value varies individually according to the given crane, whereby it depends on the manufacturing inaccuracies concerning the construction of the crane, crane track, and setting of the drives as well as on the crane span, size of the wheels and the crane track rails used.

## Data Availability

Not applicable.
